# Supplementary material for: Salivary miRNA Profiles in COVID-19 Patients with Different Disease Severities
Source: Int J Mol Sci. 2023 Jul 1;24(13):10992. doi: 10.3390/ijms241310992 (PMC10341682; doi:10.3390/ijms241310992)
Supplement: Supplementary file 1 [file ijms-24-10992-s001.zip › ijms-2441082-supplementary.pdf]

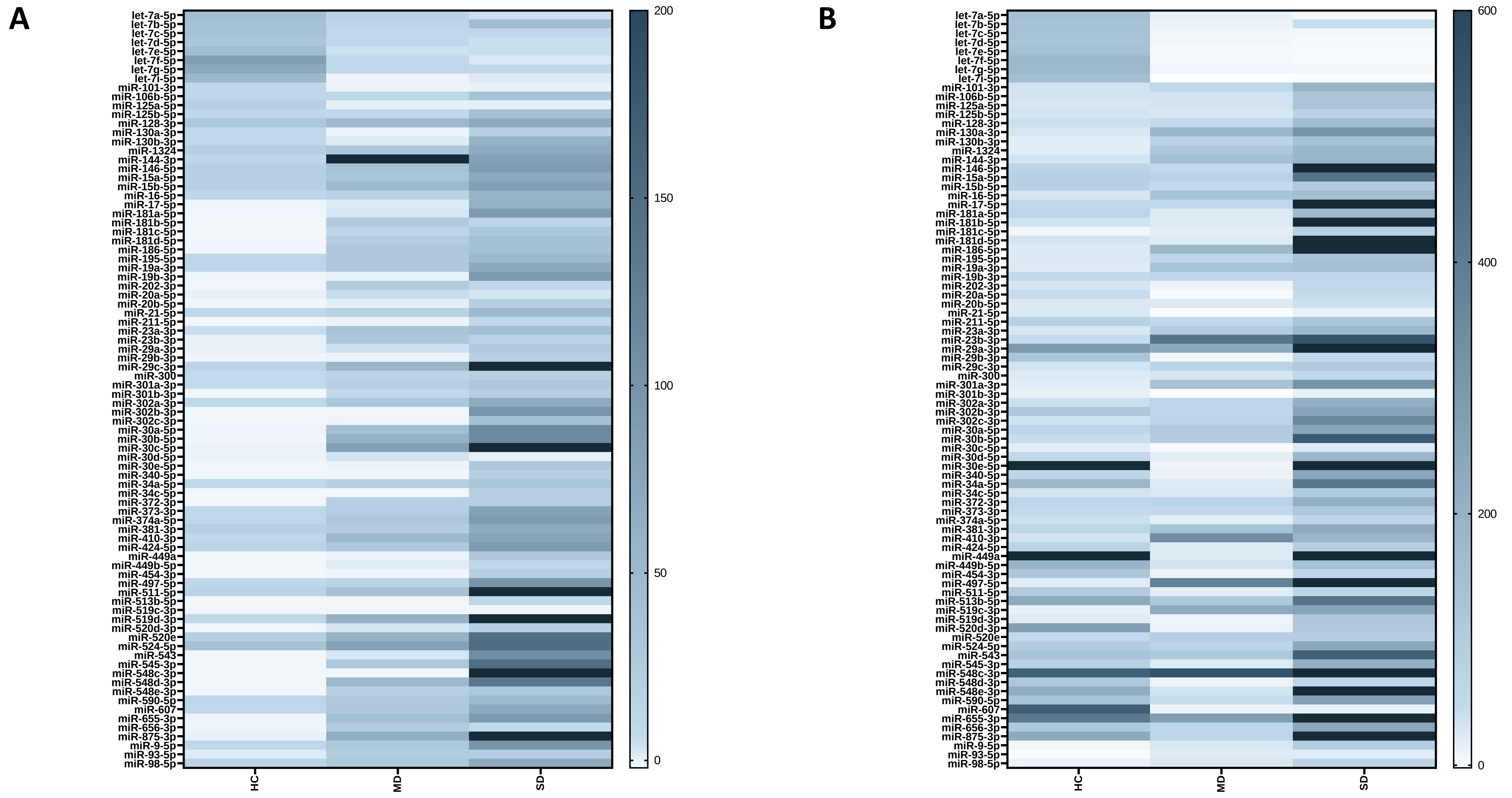

Figure S1. miRNa profile of HC, MD and SD in saliva (A) and plasma (B) samples. miRNA expression (mean values) is shown as a color scale from light blue to blue (Heatmap). Cytokines/chemokines production (mean values) is shown as a color scale from white to blue (Heatmap)
